# Identifying seasonal mobility profiles from anonymized and aggregated mobile phone data. Application in food security

**DOI:** 10.1371/journal.pone.0195714

**Published:** 2018-04-26

**Authors:** Pedro J. Zufiria, David Pastor-Escuredo, Luis Úbeda-Medina, Miguel A. Hernandez-Medina, Iker Barriales-Valbuena, Alfredo J. Morales, Damien C. Jacques, Wilfred Nkwambi, M. Bamba Diop, John Quinn, Paula Hidalgo-Sanchís, Miguel Luengo-Oroz

**Affiliations:** 1 Universidad Politécnica de Madrid, Madrid, Spain; 2 Université Catholique de Louvain, Louvain, Belgium; 3 United Nations World Food Program Senegal, Dakar, Senegal; 4 Centre de Suivi Écologique, Dakar, Senegal; 5 Pulse Lab Kampala, United Nations Global Pulse, Kampala, Uganda; CSIRO, AUSTRALIA

## Abstract

We propose a framework for the systematic analysis of mobile phone data to identify relevant mobility profiles in a population. The proposed framework allows finding distinct human mobility profiles based on the digital trace of mobile phone users characterized by a Matrix of Individual Trajectories (IT-Matrix). This matrix gathers a consistent and regularized description of individual trajectories that enables multi-scale representations along time and space, which can be used to extract aggregated indicators such as a dynamic multi-scale population count. Unsupervised clustering of individual trajectories generates mobility profiles (clusters of similar individual trajectories) which characterize relevant group behaviors preserving optimal aggregation levels for detailed and privacy-secured mobility characterization. The application of the proposed framework is illustrated by analyzing fully anonymized data on human mobility from mobile phones in Senegal at the arrondissement level over a calendar year. The analysis of monthly mobility patterns at the livelihood zone resolution resulted in the discovery and characterization of seasonal mobility profiles related with economic activities, agricultural calendars and rainfalls. The use of these mobility profiles could support the timely identification of mobility changes in vulnerable populations in response to external shocks (such as natural disasters, civil conflicts or sudden increases of food prices) to monitor food security.

## Introduction

Measuring human mobility is critical to understand population wellbeing. Mobility is characteristic of how people live and how people react and adapt to external conditions and events including climatic, social, economical or political factors [1]. Mobility is key in some regions where the agricultural production has been adapting to the changing environment. Population mobility patterns change in reaction to spatial and time variability of rainfall, or are modulated by the increasing attraction of cities compared to rural regions. At the same time, the international community has made a call for the use of new data sources and analytical methodologies to implement the new sustainable development agenda and support humanitarian action in what has been called the *data revolution* [2]. Recent developments in the scientific community and new sources of empirical social data (eg. social network platforms, applications using geolocation or mobile phone records) have enabled high spatial and temporal resolution analysis of human mobility [3–6].

The application of such type of analyses to support sustainable development and humanitarian action offers innovative solutions to existing challenges, where the access and use of data on human mobility represents an extraordinary opportunity to support programmes and policies with relevant information. For instance, social groups with different socio-economical conditions are prone to generate different mobility patterns to external shocks such as food crises which may reveal their coping strategies.

Due to the worldwide extensive penetration of mobile phones, information extracted from aggregated mobile phone meta-data has shown to be useful for development and humanitarian applications such as to model quantify and predict patterns of disease outbreaks (eg. cholera or malaria) [7, 8], understand social crises and riots [9] or natural disasters [8, 10]. The value of this data for emergency services in the aftermath of an earthquake has been shown in different contexts and geographies including Haiti [8, 11], Nepal [12], Mexico [13] or Japan [14]. Mobile phone data analysis can help to develop early warning mechanisms as well as to estimate the response of the population to external shocks in order to improve humanitarian action based on real time and accurate data [15].

The movements of a population also reflect other organizational aspects such as the population livelihoods, coping strategies and social safety nets [16]. The characterization of these social phenomena requires the tracking of migrants over sufficiently long periods of time (in the present application,the data availability allows a one year range analysis). Since this type of information may be sensitive, specific privacy-preserving schemes are also required to minimize individual details by optimizing the aggregation level and avoiding potential risks such as de-identification [17–19]. Aggregation schemes of mobility based on *Origin-Destination (OD)* matrices [20] have been extensively used, but aggregation of users’ activity prevents from consistent observation over sufficiently long periods of time (even if OD matrices are available for different instants and/or periods of time, their respective aggregations may not correspond to the same individuals; therefore, any attempt to combine them involving the time variable would not provide meaningful population results).

Hence, in this paper we propose a framework for systematic analysis of mobility which allows its characterization with flexible time, space and population aggregation capabilities. We introduce a formalism to frame and process mobile phone data containing anonymized and aggregated geographical and temporal information. In this framework, trajectory data is organized in a matrix representation where the geospatial (2*D*), temporal (*T*) and population (*P*) variables define the main data dimensions. These data are processed (via interpolation or aggregation schemes) to create a regularized *Matrix of Individual Trajectories* (IT-Matrix), which provides geolocations (with a unified resolution) so that each column is associated to the same timestamp for all rows of users. This representation allows for a systematic and consistent rescaling and/or aggregation on the matrix dimensions to quantify characteristic multi-scale patterns and indicators of human mobility. This work also proposes the selection and unsupervised clustering of some IT-Matrix rows to extract population groups sharing similar mobility behaviors and allowing to profile mobility at different resolution levels.

We have applied this framework to understand different human mobility patterns related to livelihood zones in Senegal [21] (See S1 Fig) based on data provided in the second Orange D4D Challenge [22]. A livelihood zone is an area where people generally have the same options for obtaining food and income and engaging in trade. They are determined (see [21]) by fusing geographical and/or physical information such as land cover and land use, with other socio-economic information such as census data, population density and infrastructures. Typically, people inside the same livelihood zone share options for obtaining food, income and market opportunities. However, there are multiple population groups in the same zone with different mobility signatures. Therefore, it is of interest to understand the different group behaviors existing in the livelihood. The appropriate aggregation of the IT-Matrix reveals the characteristic signatures of the population dynamics based on time-varying population count [23] in the Senegalese livelihood zones [21]. The corresponding mobility profiles show the time relationship between population mobility and environmental conditions such as the onset of the rainy season, or other indicators such as the seasonal agriculture calendars for each livelihood zone. Available seasonal calendars [21] are estimated for an average year by combining quantitative and qualitative data analyses, based on two types of data collection methods: primary (household surveys, informant interviews, etc.) and secondary (literature review, computing available statistics, etc.). These prototypical calendars can be refined to estimate actual activity cycles using other up-to-date observed data sources such as mobility information. The present work is a step towards estimating in real time the seasonal activities that are taking place (e.g. labor migration levels in the non-planting or harvesting seasons). Such analysis and insights could be relevant to derive food security monitoring indicators and to inform food security analysis and assistance targeting.

## Materials and methods

### A framework to select and cluster mobility profiles

**Fig 1:**
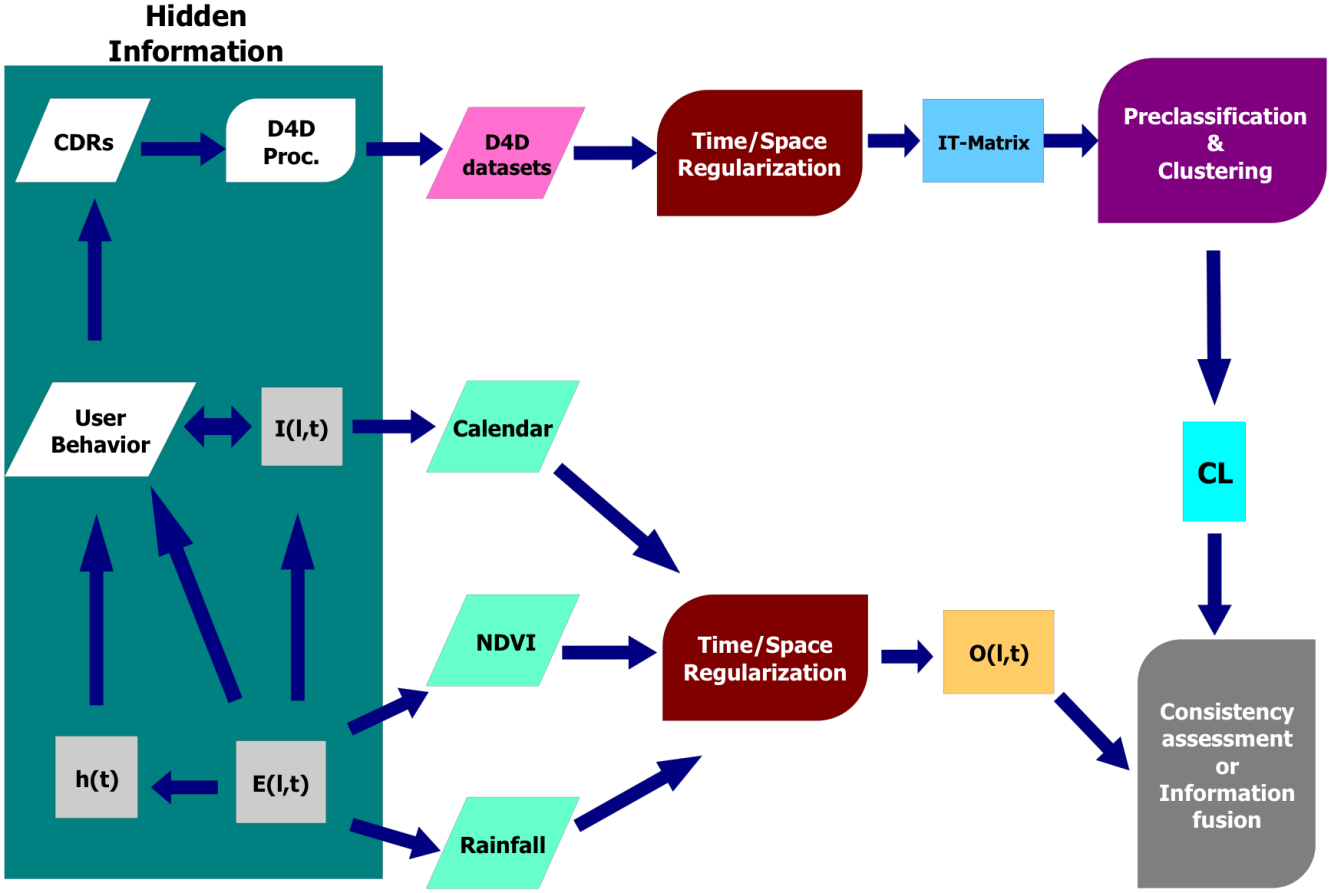
Data processing work-flow. D4D Dataset is processed for computation of IT-Matrix, which is processed to select and cluster mobility profiles. Finally, consistency assessment/fusion of profiles with other sources of data, *O*(*l*, *t*) (which depends of the *l* location and *t* time variables), such as livelihood calendars provided by WFP (World Food Program), NDVI (Normalized Difference Vegetation Index [24]) and Rain variables. Dark green rectangle represents information sources which are hidden or unknown in this study: raw Call Detail Records (CDRs); *h*(*t*), user home location; *E*(*l*, *t*), other external directly measurable variables (such as evolution of crops); and *I*(*l*, *t*), other social indicators (such as market prices).

We propose a general framework (see Fig 1) to organize and classify human mobility patterns based on individual trajectories constructed from mobile phone data. This type of data is gathered and stored by cell phone carriers (in our case, Sonatel) during communication events between their clients, providing the so called Call Detail Records (CDRs).

#### Mobile phone Data Sets

Call Detail Records conform a digital fingerprint of both the communication actions and the approximate geolocation where the events took place. Thus, this type of data has been considered a valuable source to understand human behavior such as social interactions (modeled by social networks) and mobility [3, 25]. Since analyzing CDRs entails the risk of violating users’ privacy, this data requires to be anonymized, aggregated and sometimes also quantized and/or time range limited before being used for social and public purposes. Anonymization encripts users’ personal identifiers from the CDRs whereas aggregation, quantization and range limitation prevent the identification of individuals from their behavioral patterns [18, 19].

The D4D Challenge initiative released mobile phone Data Sets (DS), derived from the CDRs of the main provider in Senegal, containing information of the location, timestamp and numeric identifiers for anonymized users’ references associated with each record from a mobile phone of the operator [22]. This data does not gather overseas visitors, which may be of interest (specially those visiting the country in particular months), and it corresponds to just about 1% of the total population. Still, such percentage of real measurements represents much more information than estimations made from some primary and secondary data sources years ago. The DS provided in the D4D Challenge show different aggregation levels as different approaches to deal with privacy and applicability. DS-1 provides the total number of calls which are routed through each pair of antennas every hour; hence, besides being space quantized at antenna coverage region resolution, it is time and population aggregated at the hour level. DS-2 is population disaggregated providing the antenna and time of calls (with 10 minute quantization) for a set of users, but it is time-range limited (15 days). DS-3 is also user disaggregated and provides 10 minute time quantized call records for a whole year, but it is space-wise quantized at a coarser set of geographical regions called *arrondissements* (as of 2013 there were 123 of them [22], each one gathering several antenna coverage regions). Other initiatives have also released mobile phone data based on different aggregation strategies such as gridded and aggregated mobile phone data descriptors [26].

Optimal aggregation level and strategy depend on the application and risk [19]. In this work, D4D DS-3 was employed as it offered one year mobility at the cost of a coarse spatial resolution—Senegalese arrondissement -. Concretely, DS-3 provides the geolocation (denoted by the 2-dimensional variable 2*D*) corresponding to *N* = 146,352 phone users (belonging to population set *P* = {1, …, *N*}) for a whole year (where the set of all possible time values in such range is denoted by *T*) with geospatial quantization into *r* = 123 disjoint *arrondissements* *R*_*i*_, *i* = 1, …, *r*. Hence, the number of levels (i.e., the resolution) on the 2*D* and *P* variables is fixed (*r* and *N*, respectively), whereas resolution along the *T* variable changes from one user to another. As shown later, information in the 2*D* × *T* × *P* space will be regularized, quantized or aggregated into different geolocation×time×population levels, allowing a multi-resolution description of mobility.

#### IT-Matrices: A trajectory-based representation of mobility

In this Section we illustrate the construction of an *Individual Trajectories Matrix* (IT-Matrix) as a consistent spatio-temporal discrete representation of human mobility at the individual level. For each individual, her/his spatial location along time will be denoted as her/his trajectory. Precisely, the trajectories during the whole time-period *T* for a set *P* of N anonymized individuals, are characterized via four variables: (*la*, *lo*) ∈ 2*D* representing user’s latitude and longitude, *t* ∈ *T* representing a time instant and *p* representing the user identification. Hence, for each *p*-th population individual we can define the position vector *x*_*p*_(*t*) = (*la*_*p*_(*t*), *lo*_*p*_(*t*)) which represents her/his spatial position at time *t*.

Unfortunately, for each trajectory *x*_*p*_(*t*) only a sampled and quantized discrete version is gathered in the CDRs, conditioned by the corresponding individual phone usage. The observation of the trajectory happens at the timestamps *t*_*p*,*j*_ where *j* indexes each instant of time when the user *p* performs a call event, generating a record; this leads to a non-uniform and user-dependent sampling process. In addition, the records are registered at the communication antenna level; the antenna locations define an initial partition of the space which gets coarser in DS-3 when only the user location arrondissement is provided (each arrondissement gathers several antennas). Accordingly, a space quantization *Q*(*x*_*p*_(*t*_*p*,*j*_)) = *R*_*i*_ if *x*_*p*_(*t*_*p*,*j*_) ∈ *R*_*i*_ has been imposed, where each arrondissement region *R*_*i*_ belongs to a final partition set *R* = {*R*_1_, …, *R*_*r*_}). Hence, for each phone user we finally have the vector:

$$\begin{array}{c} q_{p}=\left[Q\left(x_{p}\left(t_{p,1}\right)\right),\ldots ,Q\left(x_{p}\left(t_{p,n_{p}}\right)\right)\right]. \end{array}$$ (1)

Obviously, the quality of the information provided by *q*_*p*_ is conditioned by the sampling rates and quantization resolutions. Depending on such factors, one may be able to compute a good trajectory estimator ${\hat{x}}_{p}\left(t\right)$ or at least to compute time quantized or aggregated versions of it. For instance, the information provided in the D4D Challenge DS-3 contains a set *q*_*p*_, *p* ∈ {1, …, *N*} as defined in Eq (1), where each vector *q*_*p*_ may have a different length given by the number of events registered for user *p*.

In general, the analysis of trajectories can be efficiently performed when event time vectors are standardized to the same length, each component representing information associated with the same time or period of time (minutes, hours, days, months, etc.). Note that trajectories of different lengths and resolutions cannot be jointly processed in a direct and efficient manner. Hence, we propose to regularize the time sequence of events for each *q*_*p*_ to the required finer time resolution, so that we obtain a time series *z*_*p*_ which has the same length *N*_*T*_ (number of records along time) for every *p*. When the desired time scale resolution is coarser than the available data in *q*_*p*_, a sub-sampling scheme must be implemented. For instance, a temporal window (according to the desired time resolution) can be employed so that the most frequent location within the window is selected (loosely speaking this may be called a time aggregation procedure). Alternative application dependent procedures can also be employed for this aggregation [27, 28]. On the other hand, if the desired time resolution is finer than the available data, some interpolation-based scheme is required. For instance, if a user does not have any registered events in a period of time, the geolocation value of the closest previous active period of time may be assigned. In this work a regularization to daily resolution will be performed which implies estimating a daily user preferential location, providing vectors of length 365. This is motivated, as mentioned above, by the fact that trajectories of different lengths and cannot be jointly processed in an efficient manner. As it will be shown below, this time regularization will neither miss much information (when sub-sampling) nor add a significant percentage of spurious data (when interpolating). Note that both procedures tend to neglect short time-scale movements which may not be relevant for our mobility analysis purposes.

Concerning space resolution, quantization of *x*_*p*_ into *q*_*p*_ (or *z*_*p*_ if time regularization has been previously performed) is usually based on the definition of different geographical regions *R*_*i*_ ∈ *R* so that a single variable (or label) characterizes the quantized information. Furthermore, regions in *R* may be aggregated into larger areas which represent a coarser space quantization *Q*, by simply substituting each *R*_*i*_ by the larger region *Q*_*i*_ which contains *R*_*i*_. Note that such aggregation may be performed before or after the time regularization. In general, time regularization and space aggregation can be combined in different ways, and the order in which they are applied may lead to alternative time/geolocation characterizations.

In this work, time regularization at the desired resolution was performed first; then, a space quantization at the desired level was carried out. Note that if space quantization can be modeled by a single variable, the whole set of trajectories for all users can be represented via a *N* × *N*_*T*_ *Individual Trajectories Matrix* (IT-Matrix), where each row defines an individual user trajectory, each column represents the time instant (or period), and each value indicates the corresponding space location. If the range of values of such space location is also reduced to a finite set of size *N*_*D*_, the same information could be represented via a 3D binary tensor of size *N* × *N*_*T*_ × *N*_*D*_.

The high time resolution (in our case, daily) IT-Matrix allows for a straightforward construction of the IT-Matrices corresponding to coarser time resolutions. This can be done, for instance, by assigning to the new coarser time period (week, month, etc.) the most frequent value found in the days corresponding to such span of time. In our case, besides daily resolution, weekly, bi-weekly and monthly resolutions were also performed (leading to row vectors of length 365, 53, 24 and 12 respectively). For the sake of brevity, the highest resolution IT-Matrix will be simply referred to as the IT-Matrix, whereas the lower resolution IT-Matrices will be referred to as lower resolution matrices.

**Fig 2:**
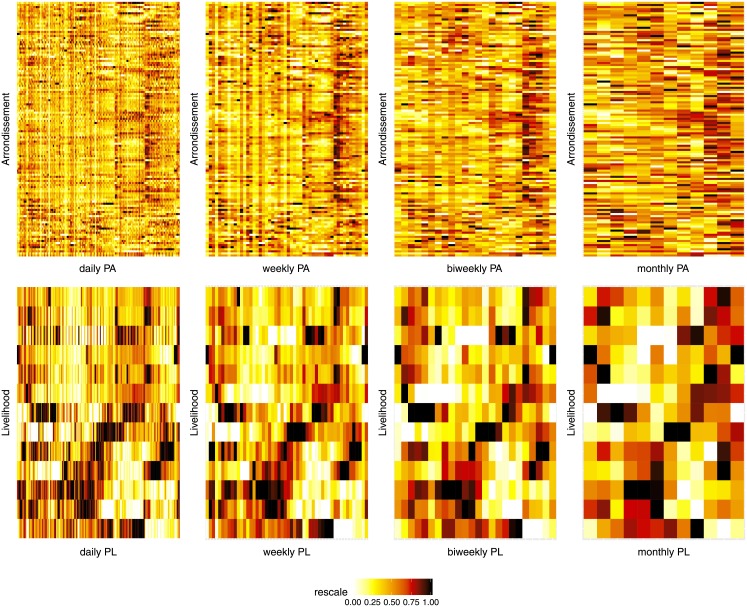
Multi-resolution population counts normalized within each region (derived from IT Matrix). First row: preferential arrondissement (PA): column 1, Daily PA; column 2, Weekly PA; column 3, Biweekly PA; column 4, Monthly PA. Second row: preferential livelihood (PL): column 1, Daily PL; column 2, Weekly PL; column 3, Biweekly; column 4, Monthly PL. Color intensity (from black to white) reflects normalized population count within each region.

The IT-Matrix and the lower (time and space) resolution matrices which can be derived from it provide estimates of the user preferential location with different time and geographical resolutions, whose information is crucial for many types of applications, including the one illustrated in this paper. For instance, daily regularization provides for each user *p* the daily preferential arrondissement (DPA) which can be used as an estimation of the daily *home location h_p_*. In general, the classical problem of estimating this latent variable *h*_*p*_ can be addressed using different schemes such as the simple computation of the most visited location during the whole day (employed in this work). For populations with high phone activity, such estimates can be refined when considering the specific hour at which the user visits each location (e.g., periods from 7pm to 7am are more likely to correspond to home location) [27], [28]. Similarly, for the application presented in this paper, a monthly regularization provides a monthly preferential arrondissement (MPA) estimate for each user. The upper row in Fig 2 shows different resolution IT-Matrices of the different temporal aggregations for the arrondissement spatial resolution (visualization has been qualitatively improved by normalizing the color scale within each arrondissement). In addition, using the D4D contextual data (in shapefile format) to aggregate the population from 123 arrondissements to 13 livelihoods zones [21], as illustrated in S2 Fig, allows to obtain the convenient monthly preferential livelihood (MPL) zone for each user. The lower row of Fig 2 illustrates the IT-Matrices of the different temporal aggregations for the livelihood spatial resolution (again the color scale has been normalized within each livelihood).

#### Trajectory selection: Geolocation and basic mobility properties

Aimed to analyse human mobility between different regions, a first stage selection of users can be performed, based on both the regions they visit and some basic predefined mobility properties. Although the results of this selection may depend on the time and space resolutions employed, here monthly resolution time series and livelihood (or arrondissement) zones will be considered to illustrate the proposed methodology. If we consider all users who have visited a given livelihood zone for some month (first selection criterion), very different behaviors can be found, ranging from those users who visited the livelihood zone only during one month (occasional) to users who stayed in such livelihood zone during all 12 months of the year (non-moving). For each livelihood zone, a histogram can be computed to represent the ratio of visitors as a function of the number of months stayed in such livelihood zone (see S3 Fig). This information allows to quantify the proportion of people that are removed when performing any population selection based on their mobility profiles.

For example, since we are targeting some specific moving users, one can remove non-moving users. In addition, in order to detect unusual movements, if we are given an IT-Matrix with arrondissement space resolution, users for which the geographical distance corresponding to their change of arrondissement does not surpass a given ratio (e.g. 3) with respect to their radius of gyration (an estimate of the user expected moving distance which can be obtained from the bandicoot toolbox [29]), can be also removed as “regular travelers”. The distance corresponding to an arrondissement change was computed as the distance between the respective centroids; hence, this selection is very sensitive to the size of the involved arrondissements. Finally, users belonging to arrondissements labeled as urban areas (e.g., based on night-time light levels obtained from Satellite Data [30]) may also be removed if required.

When targeting a specific livelihood zone (L), several parametrized temporal constraints motivated by our final objective can be used for further selection such as: 1) the user must have stayed at least a given minimum number of consecutive months in the target L; 2) the user must have not stayed more than a given maximum number of months in the target L; 3) the user must have stayed at least another given minimum number of months in some other L; and 4) the user must have stayed in L at a specific period of the year (when looking for specific types of mobility profiles, such as the ones related to agricultural events). The selection parameters can be chosen keeping in mind the percentage of discarded people so that the representativeness of the moving population is ensured (see S3 Fig). In the following, a final stage classification of the selected mobility profiles is presented.

#### Final classification of mobility profiles via unsupervised clustering

The final step of the analysis is aimed to divide the initial dataset containing the whole population into different population groups, where each group has a distinctive mobility pattern. For this purpose an unsupervised clustering algorithm will group together the individuals represented by their mobility profiles. Note that when individuals are characterized by their movements at different time and/or space resolution levels, the resulting population subgroups with a distinctive mobility pattern may change. Since IT-Matrix gathers a heavily quantized space description of mobility behavior (both before and after binarization), its simplified structure allows for a direct application of classical clustering schemes to the mobility profile vectors, avoiding the use of specific tools for time series clustering [31, 32].

Different time and/or space resolution levels may lead to different clustering or partition outcomes. The appropriate time scale (monthly, biweekly, weekly, daily) is determined by the type of mobility patterns we want to detect and by the computational limitations inherent to clustering techniques for high-dimensional data [33–35]. In order to detect seasonal livelihood related behaviors such as workforce flows to balance urban and rural jobs, monthly or biweekly time resolutions seem appropriate. Note that a weekly resolution may capture quite ephemeral movements for our purpose and it leads to high dimension (54 weeks) vectors.

We illustrate the processing scheme only for monthly resolution profiles representing livelihood zones, since the objective is to characterize seasonal behaviors related to the livelihood’s production means and coping strategies. The resulting MPs are integrated with other sources of data (to be considered below) also provided with these levels of time resolution. Hence, *N*_*T*_ = 12 so that the corresponding IT-Matrix will be composed of *N* rows of 12-dimensional vectors whose components provide the monthly preferential livelihood (MPL) of the corresponding user.

The selection stage outputs a set of users who have visited each livelihood zone as moving population; then, their MPL are binarized to simply indicate for each month if the user is or is not in the livelihood zone under consideration. These binary mobility profiles serve as a simplified and normalized sub-matrix for each livelihood zone that can be classified into different groups attending to their similarity, allowing interpretations about the inwards and outwards mobility across livelihoods. For instance, one can observe a significant population decline during rainy season on the eastern livelihoods of Senegal.

In order to classify the selected and binarized MPL, different clustering schemes were considered depending on the type of distance defined between vectors and the clustering procedure. In our application, Jaccard distance [36, 37] was selected as the most appropriate for quantifying the targeted similarity between binary patterns. Hierarchical clustering (most suitable for binary vectors) was finally employed, where different distance criteria between groups (Ward, average, complete) provided similar results. The resulting dendrogram tree can be cut by a maximum number of representative classes for each livelihood zone, where each cluster stands for a mobility profile class within the population that has occupied the target livelihood under the imposed constraints.

The different clusters of trajectories provide consistent mobility profiles in the population. These profiles may be fundamental to understand social behaviors (e.g., to outline socio-economic profiles) and to characterize population movements due to seasonal changes or large scale events. In the following Section we analyse the relationship between these profiles and other measurements such as external variables or social indicators.

### Integration of other data sources for assessment of consistency and contextual analysis

As a complement to the information derived from the CDRs, there are additional variables which can be estimated from other data sources, and which may serve for checking the consistency of our analysis and/or for characterizing their effect in user mobility. These variables can be classified into *External variables*, *E*(*l*, *t*), and *Indicators*, *I*(*l*, *t*) (see Fig 1). External variables refer to directly measurable variables which affect user behavior and depend on geographical location *l* and time *t*, such as rainfalls, evolution of crops (e.g., variables derived from NDVI), holiday calendars, etc. gather other human derived variables with social information which may be relevant for the work objective, such as source income calendars, market prices (in location *l* at time *t*), or measurements related to food security as the food consumption score which measures diversity and frequency of food groups consumed by populations [38].

Although the information provided by mobility profiles has usually different resolution scales (richer in time and coarser in space) than the information available from external variables or indicators, such profiles and/or those variables or indicators can be filtered and/or projected (via appropriate quantizations or aggregations on the 2*D* × *T* × *P* space) to compute correlations among them, as a way to partially assess the consistency of the profile analysis (see again Fig 1).

### Software tools

In the course of this work a Spark cluster with a HDFS storage system has been employed to digest and analyze the data with pySpark [39]. Clustering of mobility profiles has been performed with the pySpark MLlib library [40], while preliminary analysis was performed using the corresponding R libraries [41].

## Results

### Multi-resolution dynamic population count from IT-Matrices

We used the D4D DS-3 dataset (see Materials and methods) to build the highest resolution IT-Matrix containing complete trajectories of *N* = 146,352 users during 2013. The trajectories were regularized from the original time resolution of 10 minutes to obtain a daily location along the year, comprising the aggregation of daily activity and the interpolation of missing days. Thus, the derived highest resolution IT-Matrix discriminates among *N*_*D*_ = 123 Senegalese arrondissements, with a daily resolution, leading to the daily preferential arrondissement (DPA) IT-Matrix of size 146,352 × 365 × 123.

As mentioned above (Materials and methods), different mobility patterns can be detected depending on the time resolution employed; hence, such time resolution will be selected according to the targeted objectives and the intrinsic limitation of mobile data (e.g. time between calls). In our case, since we are characterizing agriculture related movements at coarse spatial resolution and we are interested in seasonal mobility, we estimated appropriate to select bi-weekly and monthly resolution: there is enough signal in the mobile data and capturing mobility phenomena few weeks in advance or in retard could support implementing policy and programmes. The DPA IT-Matrix was further quantized in time by computing the most used location by month to generate the biweekly and monthly (the relevance of these two time resolutions has been explained above) preferential arrondissement (BPA and MPA) IT-Matrix of size 146,352 × 24 (or 12 resp.) with values in {1, …, 123}. In addition, we quantized the geolocation by assigning each arrondissement to its corresponding livelihood zone (see S1 and S2 Figs and S1 Note for details). This geographical transformation enabled for an agriculture related characterization of the users’ geolocation, reducing also the dimensionality to obtain the DPL, BPL and MPL IT-Matrices of size 146,352 × 365 (or 24,12 resp.) with values in {1, …, 13}. Thus, the systematic construction of IT-Matrices led to a multi-resolution representation of human mobility in Senegal for 2013.

Provided a temporal regularization of trajectories, each IT-Matrix implicitly defined a consistent dynamic population count at the corresponding geographical and temporal resolutions.

Fig 2 shows dynamic population counts as 2*D* images with hot colormaps. The spatial quantization filtered out local patterns of each specific arrondissements preserving main landmarks and abrupt changes along time (figures first row vs. figures second row). The temporal quantization (figures first column vs. figures second, third and fourth columns) removed the short-time landmarks while keeping changes between longer periods (weeks, biweeks and months). A spectral analysis of daily IT-Matrix sequences shows that low frequencies prevail so that time quantization preserves most of the information (specially the seasonal-related one we are interested in). The temporal and spatial quantized population count from the MPL IT-Matrix provided a simplified image of the seasonal population movements with distinctive patterns for each livelihood zone (L).

**Fig 3:**
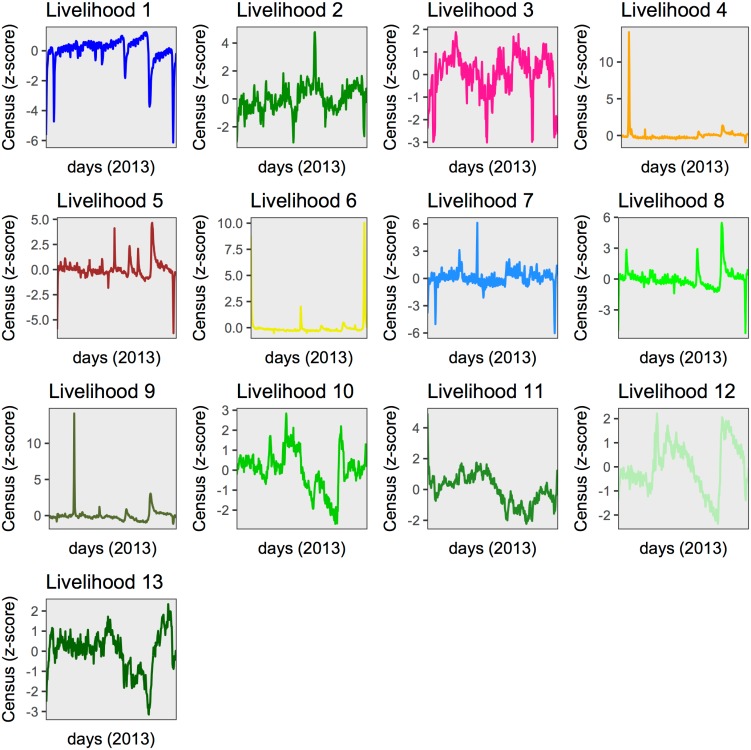
Dynamic population counts obtained from regularized DPL of IT-Matrix. Line colors follow the color code of livelihood zones maps in S1 Fig.

The MPL IT-Matrix was used to further understand the human mobility flows in the country, since it provides a monthly state of the population distribution in the livelihoods together with the temporal evolution of the livelihoods occupancy. This spatio-temporal resolution was convenient to understand seasonal migration related to agricultural calendars and represent the migration in terms of month-livelihood population count. The count variations represented by their z-score provided the normalized seasonal signatures for each livelihood zone (Fig 3). These signatures were used to assess the impact of the interpolation of missing days by the IT-Matrix temporal regularization (Materials and methods). Considering that the D4D DS-3 was already filtered to contain users that were active at least for 256 days during 2013 [22], the possible distortion caused by interpolation was initially limited by the D4D Challenge protocols.

We compared the population count of the DPL IT-Matrix with the non regularized version of it to assess the effect of interpolating missing days of activity in the original DPA IT-Matrix in terms of the added users by day in each livelihood by the interpolation (Fig 3 and S4 Fig). The difference between the signatures indicated that the mean bias effect of the interpolation was upper limited to a 8 − 10% (S4 Fig); this bias happened mostly in the first two months of the year due to the lack of previous temporal information, so that missing data in the initial days of the year was filled up backwards with the recorded value of the first active day (S4 Fig). However, the variations of both population counts showed similar trends and peaks as illustrated in Fig 3. As mentioned above (Materials and methods) such interpolation of missing days was performed by using the last known position of the user. Note that for non-interpolated data valleys in the population count of a given livelihood zone are associated with either temporary suspension of communication activity or a temporary displacement to another livelihood zone; the interpolation scheme would fill such valleys assuming the user stays in the same livelihood zone unless he/she moved and called from other livelihood zone. The gradients observed in both population counts (non-interpolated and interpolated) suggest that indeed users made calls when leaving their livelihood zones. Similarly, peaks were preserved so that short-term mobility due to holidays was also captured since there were calls before, during and after the event. Hence, the bias introduced by the regularization of the trajectories for the D4D DS-3 was considered negligible to study patterns of seasonal mobility.

The DPL dynamic population count (Fig 3) revealed both short-term events and longer-term trends. Regarding yearly trends, it is observed that most rural livelihood zones (L 10-13) suffered a severe population reduction during summer. On the other hand, Dakar (L 1) showed an increase of population during summer and a sharp gap by the end of it, suggesting that it attracts rural population during such period of the year. Livelihood zones 2-7 showed stationary patterns mainly characterized by peaks corresponding to holidays. Other short-term events were significant in livelihood zones 8 and 9 where one can see landmarks as peaks of high population at the beginning and end of this season corresponding to holidays.

### Seasonal human mobility patterns in livelihood zones through mobility profiles

#### Representativeness of moving population

**Fig 4:**
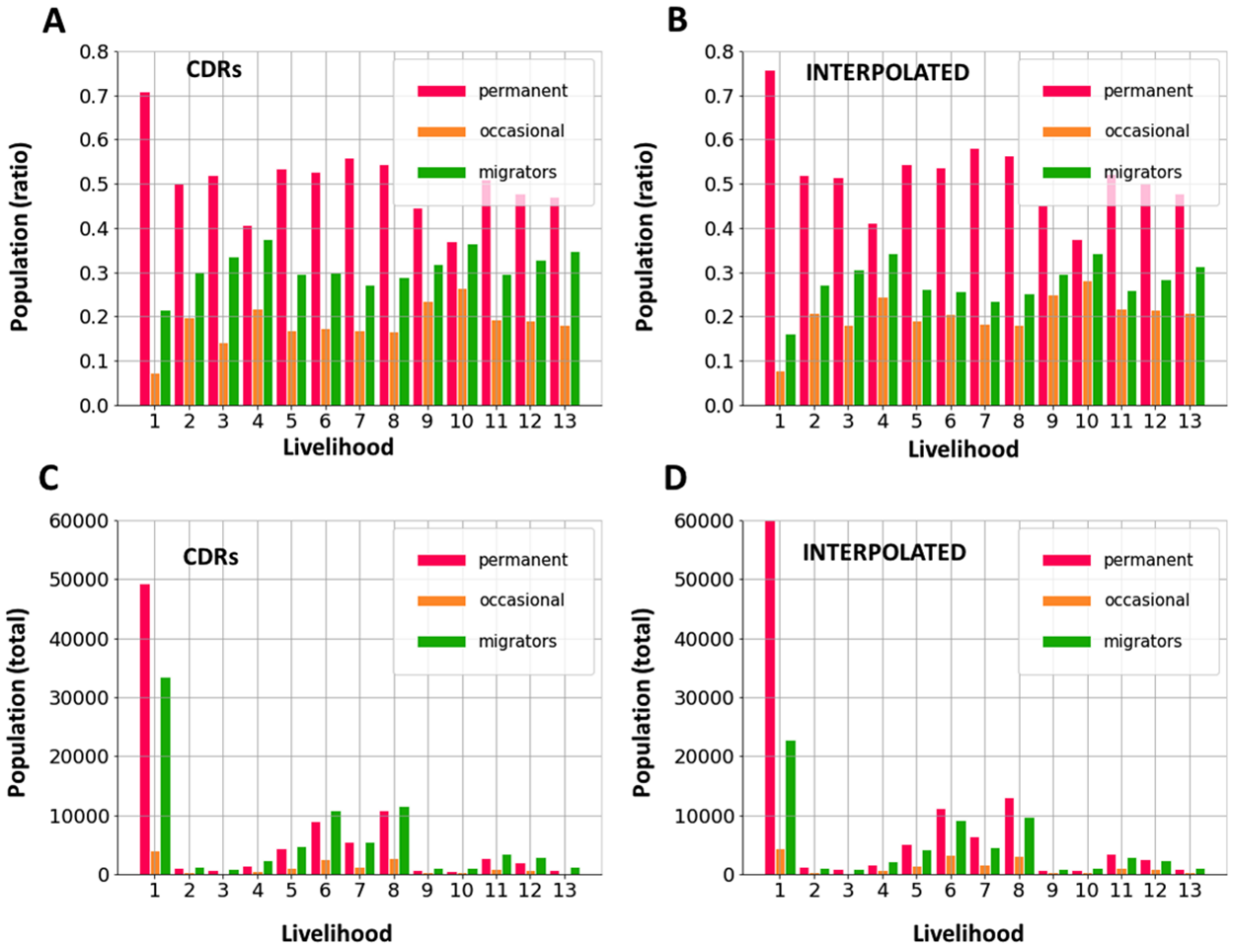
Representativeness of moving population. Permanent (non-moving), occasional visitors (1 month) and moving/migratory (2 to 11 months).

Provided a constant total population due to the regularization, the counts were assumed to be modulated by population movements. For characterizing the moving population ratio, different selection or filtering criteria might be applied (Materials and methods). We explored the distribution of users’ occupancy in each livelihood zone (S3 Fig). This distribution showed higher concentration of very short-time visitors or permanent residents and a more spread mid-term population that contributed to the modulation of the dynamic population count. Therefore, we applied a simple threshold-based selection of the population: occasional visitors in a livelihood zone (1 month) contributed to a 10% of the dynamic population, while permanent residents (12 months) conformed a 40 − 50% in most of the livelihoods and around a 70% of the population in Dakar (livelihood 1 in Fig 4). Moving/migratory population (2 to 11 months) represented the remaining 40 − 50% of the population of rural areas (livelihood zones 2-13—Fig 4).

Moving visitors in Dakar represented a 25% of the population in the city, which is still larger than the total population of any livelihood zone (Fig 4). The regularized trajectories showed more percentage of permanent residents than the original data, implying that the (interpolated) missing days in CDRs will more likely correspond to users with scarce mobility. This conclusion supported the results from Fig 3 and the previous assumption of the bias introduced by temporal interpolation being negligible.

#### Mobility profiles of livelihoods

**Fig 5:**
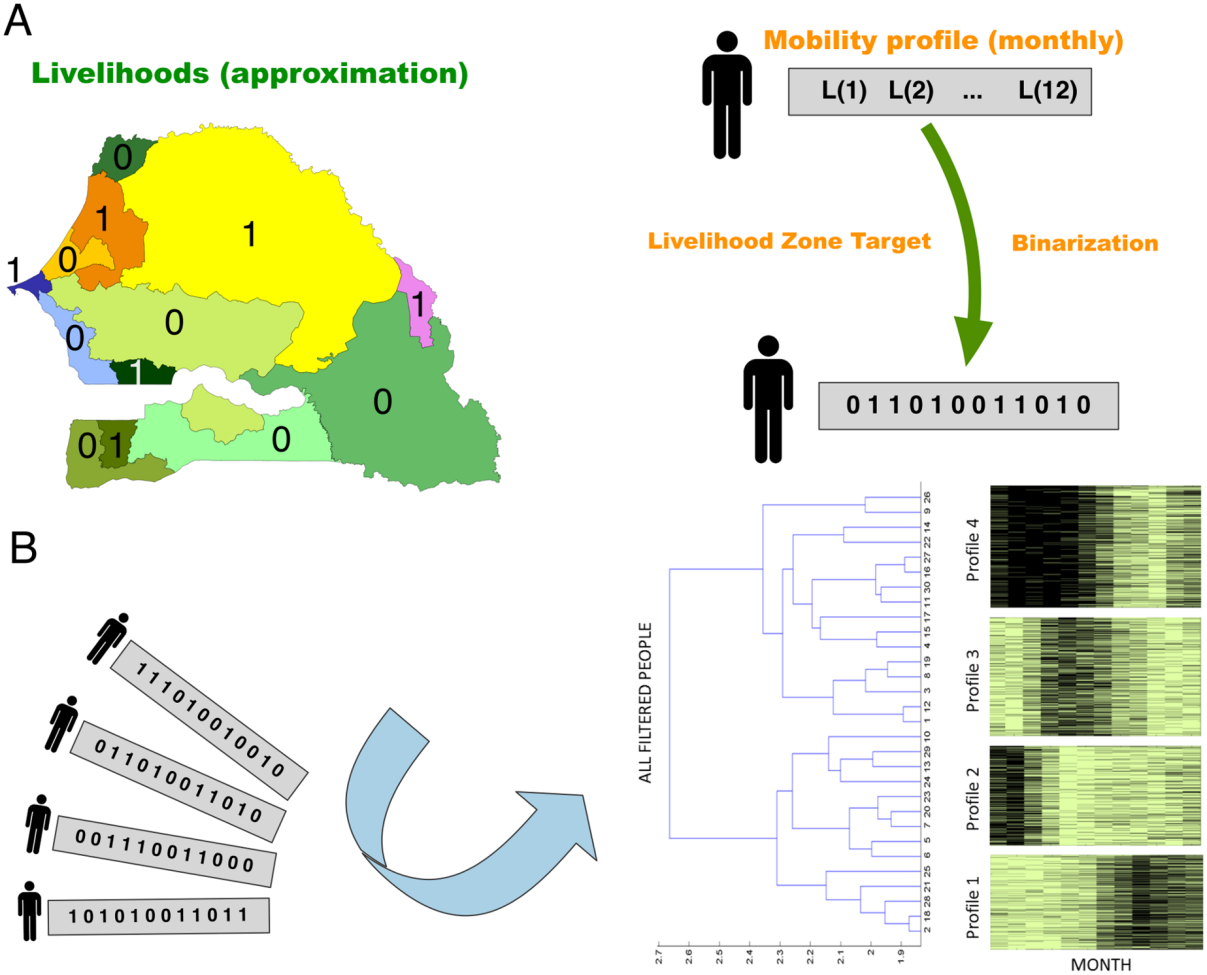
Classification of moving population. A) Binary codification of IT-Matrix (detailed user trajectories) corresponding to each livelihood zone. B) Hierarchical clustering based classification of binary vectors providing the relevant groups of mobility profiles corresponding to each livelihood zone.

The classification by mobility profiles (MPs) of the moving population in the livelihood zones (2-11 months of occupancy) was performed to quantify the diversity of mobility patterns (see Materials and methods). The scheme of the process to obtain the MPs is shown in Fig 5. The classification of the binary IT-Matrix corresponding to each livelihood zone into *k* mobility profiles provided *k* dynamic population counts within the livelihood zone (Fig 5 illustrates the case for *k* = 4). Several clustering techniques combinations were compared, the results being less sensitive to such techniques than to the employed time resolution.

#### Mobility profiles and agriculture monitoring

**Fig 6:**
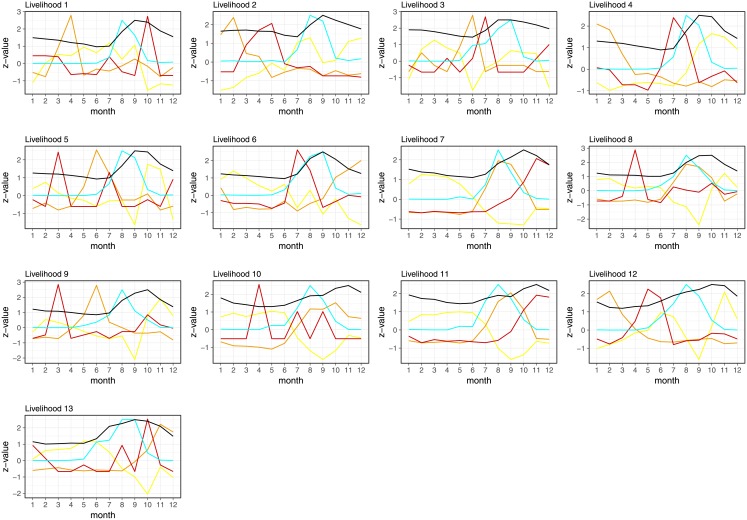
Seasonal mobility profiles in each livelihood zone. They are clustered with *k* = 3: yellow, red and orange. Each curve represents z-scored values of the population count Livelihood zones 1-13 (see S1 Fig) are ordered up-down, left-right. Cyan curves show the rainfall estimations averaged by livelihood zone, whereas black curves show the NDVI estimation averaged by livelihood zone. Both rain and NDVI curves have been rescaled to fit the scale of the population count signatures.

Mobility profiles were assessed and evaluated with external data sources of environmental and agricultural variables (Materials and methods). Satellite remote sensing is widely used in agriculture monitoring as it is particularly suitable for providing a timely and accurate picture of crop status and conditions over large areas with high revisit frequency [42]. The time series profile of the NDVI index (see S2 Note) delivered critical information on the phenological dynamics of an agricultural landscape (start of the season, peak of the season, start of senescence, etc.) [43, 44]. Rainfall estimations obtained from the NASA-TRMM project [45] data were processed and aggregated in a coarse resolution for a temporal indicator of the rainy season (see S2 Note). We integrated these variables with the mobility profiles (Fig 6, *k* = 3, yellow, red and orange) for a rich description of seasonal dynamics in the livelihood zones. Rainfalls onset and peak (Fig 6, cyan) preceded the vegetation index (Fig 6 black) whose peak was reached in the month after the peak of rainfalls. The onset of rainfalls in June-July triggered the change of the most significant MP in the rural livelihood zones indicating large outwards mobility from rural areas at the beginning of the rainy season.

Finally, correlations of the different MPs with source income calendars [21] derived from households surveys were also computed. No clear correlations were found so that the utility of such calendars as a possible ground-truth static information of the crop periods in the livelihood zones remains an open issue. Calendars corresponding to livelihood zone 6 and the corresponding obtained MP can be seen in S4 Fig. Data corresponding to further years would help to clarify this issue.

## Discussion

The proposed framework of human mobility analysis based on IT-Matrices enables for a flexible time-space characterization of seasonal or event related behaviors. This approach establishes a multi-scale framework generalizing approaches based on OD-Matrices that have been proved useful for the aggregated analysis of social events or urban transportation. The regularization procedures when generating IT-Matrices efficiently deal with missing mobility information due to non-regular use of mobile phone while preserving the main information concerning population mobility, as illustrated in the comparative dynamic population counts and statistics (Fig 4). The robustness of the mobility analysis in terms of the similarity between population counts when interpolated and non-interpolated trajectories are employed suggests that mobility and phone activity are associated: undersampled trajectories tend to correspond to non-moving population, so that most part of the population movements are observed through phone data even in rural contexts, provided that only users performing a minimum number of calls during the year are considered. However, these results may depend on the aggregation and sampling procedures applied to CDRs. Therefore, further studies with ground truth data corresponding to population movements are required to asses the minimum sampling rate necessary to properly account for people’s mobility.

Selection and unsupervised classification of the trajectories led to the definition of mobility profiles (MPs) as a privacy-safe, still detailed and descriptive strategy to disaggregate different mobility patterns. The comparative dynamics between the MPs and environmental (rainfall) and crop indicators (NDVI) allowed to quantify and interpret the mobility patterns during 2013. It is important to note that, in this work, raw data corresponds to a provider with a market share over 60% of the population, representing a quite complete dataset not as limited and biased as in other CDRs analysis projects.

The application example illustrated in this work was motivated by the need of analyzing and quantifying the role of mobility patterns in the communities lifestyles and their access to basic resources, using a more precise and up-to-date information (the one provided by the CDRs) than the ones employed previously (polls, a posteriori social indicators, etc.).

The volume and timing of the arriving and leaving workforce in agricultural areas could potentially be an interesting indicator on the expected production and harvest time. A high demand in workforce at the beginning of growing season could for example indicate in advance large (expected to be successful) planted areas, while lower workforce than usual at the end of the season could indicate lower production. Since workers try to maximize their income by balancing rural and urban jobs depending on the expecting labor demand, rural to/from urban areas mobility becomes a key indicator. In this sense, the correlation analyses performed between mobility patterns and Source Income Calendars can be used to define a baseline behavior, provided several years of data are available; this way anomalous mobility patterns could be detected almost in real time. In addition, although the antenna density may be too low for characterizing mobility around rural markets, the analysis of the access to urban centers could be an interesting alternative. Therefore, with the support of additional data, this new approach to unravel and analyze mobility patterns could be very helpful to supervise the evolution of production means of Senegal in order to monitor vulnerable communities.

One of the limitations of this study is the availability of one year of data. Studies comprising several years of data with different environmental situations are necessary to understand and define how a systematic mobility analysis could be deployed as a real-time tool for food security. Such tool could be used as an early warning mechanism via comparison with long term averages.

## Conclusion

This work shows the feasibility of processing phone data to obtain a systematic identification of population mobility profiles as a tool to understand population seasonal mobility behaviors with a flexible characterization of users mobility at different aggregation levels in both space and time. Clustering individual trajectories into different mobility profiles allows to understand how different groups of people behave at longer temporal scales (e.g, during a year) changing their place of living due to socio-economic factors and livelihood styles.

While the theoretical framework presented in this study is generic, we have illustrated the application of this methodology to the D4D Challenge in Senegal. Although mobility behaviors due to agricultural cycles and their timing are qualitatively known, there is little quantitative information on exact timing and scale of those population movements (e.g. how many people go from the city to rural areas in specific harvest seasons). Results using the proposed framework showed the potential to fill this data gap and provided relevant information related to the population activity in the different livelihood zones in Senegal allowing to measure the changes in mobility patterns related to the agricultural production means.

Generally, climate change and socioeconomic pressures are constantly changing the conditions that affect livelihoods, and complete household surveys to collect the information are a expensive and resource intensive endeavor. Therefore, always under strict and secure privacy frameworks, the aggregated analysis of populations’ mobility, could be a valuable tool to help policy makers and practitioners quantify and uncover new population movement phenomena; so better policies and social protection programs can be designed.

## Supporting information

S1 Note Livelihoods and source income calendars. (PDF)

S2 Note Normalized Difference Vegetation Index. (PDF)

S3 Note Rainfall estimations from NASA-TRMM project. (PDF)

S1 Fig Senegalese segmentation into livelihood zones. Left: Map shows the segmentation of Senegal into different livelihood zones. Right: Summary of each livelihood zone main characteristics (further information in [21]).
(EPS)

S2 Fig Senegalese segmentation into arrondissements. The color indicates the livelihood assigned to each arrondissement, which is the one with larger overlapping area with the arrondissement surface. Antenna locations are also displayed with red dots.
(EPS)

S3 Fig Length of occupation by density of population. Histograms and associated boxplots corresponding to the length of occupation of the population density in each livelihood 1-13 up-down, left-right (see S1 Fig). Blue histograms show the distribution of the raw CDRs in monthly resolution. Red histograms show the distribution of the interpolated data in monthly resolution.
(EPS)

S4 Fig Completed vs original CDR-based population count at monthly resolution. Dynamic population count for each livelihood (colors match the map in S1 Fig). They were computed by counting the users located within the livelihood shapefile. Right columns show the completed count after daily interpolation and temporal aggregation (Material and methods) and left columns show the original CDR-based count.
(EPS)

S5 Fig Mobility profiles and livelihood calendars. Alignment of the sylvo-pastoral livelihood (yellow livelihood in S1 Fig) calendar and the derived mobility profiles for the livelihood.
(EPS)
