# Supplementary material for: Identifying seasonal mobility profiles from anonymized and aggregated mobile phone data. Application in food security
Source: PLoS One. 2018 Apr 26;13(4):e0195714. doi: 10.1371/journal.pone.0195714 (PMC5919706; doi:10.1371/journal.pone.0195714)
Supplement: S1 Note — (PDF) [file pone.0195714.s001.pdf]

Supporting Information of "Identifying seasonal mobility profiles from anonymized and aggregated mobile phone data. Application in food security"

Pedro J. Zufiria[1◑*], David Pastor-Escuredo[1◑], Luis Úbeda-Medina[1◑], Miguel A. Hernandez-Medina[1‡], Iker Barriales-Valbuena[1‡], Alfredo J. Morales[1‡], Damien C. Jacques[2,‡], Wilfred Nkwambi[3,‡], M. Bamba Diop[4,‡], John Quinn[5,‡], Paula Hidalgo-Sanchís[5,‡], Miguel Luengo-Oroz[5,‡]

1 Universidad Politécnica de Madrid, Madrid, Spain
2 Université Catholique de Louvain, Louvain, Belgium
3 United Nations World Food Program Senegal, Dakar, Senegal
4 Centre de Suivi Écologique, Dakar, Senegal
5 Pulse Lab Kampala, United Nations Global Pulse, Kampala, Uganda

* Corresponding author
E-mail: pedro.zufiria@upm.es (PJZ)

◑These authors contributed equally to this work.
‡These authors also contributed equally to this work.

Supporting Information

S1 Note: Livelihoods and Source Income Calendars

The main forms of livelihood in Senegal vary from region to region, and the country can be divided into a number of zones (see S1 Fig) where, for example, pastoralism, agriculture or fishing are the dominant activities. Within each of these "livelihood zones", there are well-studied patterns of seasonal activities and population movements [1].

Source Income Calendars provided by the World Food Program [2] are a reference of the *most prevalent* activity in each livelihood zone. The correlations or discrepancies between mobility patterns and calendars may indicate influences or uncommon (and hence, relevant) behavior. The construction of a baseline along several years may help to elucidate this dichotomy.

References

1. Famine Early Warning Systems (FEWS) NET. Senegal;. Accessed: 2017-03-01. Available from: `http://www.fews.net/west-africa/senegal`.

2. WFP. Comprehensive food security, nutrition and security vulnerability, July 2014;. Accessed: 2017-03-01. Available from: `http://vam.wfp.org/CountryPage_assessments.aspx?iso3=SEN`.
