# Supplementary material for: Identifying seasonal mobility profiles from anonymized and aggregated mobile phone data. Application in food security
Source: PLoS One. 2018 Apr 26;13(4):e0195714. doi: 10.1371/journal.pone.0195714 (PMC5919706; doi:10.1371/journal.pone.0195714)
Supplement: S2 Note — (PDF) [file pone.0195714.s002.pdf]

Supporting Information of "Identifying seasonal mobility profiles from anonymized and aggregated mobile phone data. Application in food security"

Pedro J. Zufiria[1◑*], David Pastor-Escuredo[1◑], Luis Úbeda-Medina[1◑], Miguel A. Hernandez-Medina[1‡], Iker Barriales-Valbuena[1‡], Alfredo J. Morales[1‡], Damien C. Jacques[2,‡], Wilfred Nkwambi[3,‡], M. Bamba Diop[4,‡], John Quinn[5,‡], Paula Hidalgo-Sanchís[5,‡], Miguel Luengo-Oroz[5,‡]

1 Universidad Politécnica de Madrid, Madrid, Spain
2 Université Catholique de Louvain, Louvain, Belgium
3 United Nations World Food Program Senegal, Dakar, Senegal
4 Centre de Suivi Écologique, Dakar, Senegal
5 Pulse Lab Kampala, United Nations Global Pulse, Kampala, Uganda

* Corresponding author
E-mail: pedro.zufiria@upm.es (PJZ)

◑These authors contributed equally to this work.
‡These authors also contributed equally to this work.

Supporting Information

S2 Note: Normalized Difference Vegetation Index

The interest of the combination of vegetation reflectances in the red and near infrared (so called vegetation indexes or VI) for vegetation characterization is well known since the early 80s [1]. Among all the existing vegetations indexes, the Normalized Difference Vegetation Index (NDVI) is unquestionably the most popular indicator for studying vegetation health and crop production. The NDVI values typically vary from 0.15 for bare soils to 0.80 for full green vegetation, with all gradations in-between.

10-day temporal syntheses of Spot-Vegetation [2] NDVI at a resolution of 1-km per pixel have been downloaded over the area of interest for 2013. In the multi-temporal image set, each pixel is thus characterized by a specific NDVI-time profile. However, since the raw profiles are still disturbed by cloudy measurements, the composite images are first submitted to a cleaning procedure by means of the Whittaker smoother [3]. To consider only NDVI information related to agriculture, cropland areas were masked using the Senegal Land Cover Map of 2005 at the 1 : 100.000 scale produced by the Global Land Cover Network [4]. Finally, pixels' values of each livelihood zone were aggregated to provide mean and standard deviation of NDVI for each zone and each month. Average mean NDVI values per month (averaged all over the country) ranged from 0.224 in May to 0.454 in October, with average standard deviations ranging from 0.048 to 0.1; average mean NDVI values per livelihood (averaged the whole year) ranged from 0.216 in L 3 to 0.466 in L 10, with average standard deviations ranging from 0.03 to 0.16.

References

1. Tucker CJ. Red and photographic infrared linear combinations for monitoring vegetation. Remote sensing of Environment. 1979;8(2):127–150.

2. SPOT Vegetation Program.;. Accessed: 2017-03-01. Available from: www.vgt.vito.be/.

3. Eilers PH. A perfect smoother. Analytical chemistry. 2003;75(14):3631–3636.

4. Leonardi U. Senegal Land Cover Mapping. Food and Agriculture Organization of the United Nations; 2008.
