# Supplementary material for: Identifying seasonal mobility profiles from anonymized and aggregated mobile phone data. Application in food security
Source: PLoS One. 2018 Apr 26;13(4):e0195714. doi: 10.1371/journal.pone.0195714 (PMC5919706; doi:10.1371/journal.pone.0195714)
Supplement: S3 Note — (PDF) [file pone.0195714.s003.pdf]

Supporting Information of "Identifying seasonal mobility profiles from anonymized and aggregated mobile phone data. Application in food security"

Pedro J. Zufiria[1◑*], David Pastor-Escuredo[1◑], Luis Úbeda-Medina[1◑], Miguel A. Hernandez-Medina[1‡], Iker Barriales-Valbuena[1‡], Alfredo J. Morales[1‡], Damien C. Jacques[2,‡], Wilfred Nkwambi[3,‡], M. Bamba Diop[4,‡], John Quinn[5,‡], Paula Hidalgo-Sanchís[5,‡], Miguel Luengo-Oroz[5,‡]

1 Universidad Politécnica de Madrid, Madrid, Spain
2 Université Catholique de Louvain, Louvain, Belgium
3 United Nations World Food Program Senegal, Dakar, Senegal
4 Centre de Suivi Écologique, Dakar, Senegal
5 Pulse Lab Kampala, United Nations Global Pulse, Kampala, Uganda

* Corresponding author
E-mail: pedro.zufiria@upm.es (PJZ)

◑These authors contributed equally to this work.
‡These authors also contributed equally to this work.

Supporting Information

S3 Note: Rainfall estimations from NASA-TRMM project

Daily Rainfalls at different geographical resolutions were obtained from a geographical aggregation of NASA's TRMM sensed data [1], collected with a 0.25 resolution (longitude and latitude) in a daily basis. The average precipitation by Arrondisment in 2013 and the corresponding standard deviation are 2450 mm and 1483 mm respectively. The raster data was segmented using the livelihoods shapefile calculating the average rainfall estimation by livelihood [2].

References

1. Goddard Earth Sciences Data and Information Services Center(2016), TRMM (TMPA) Precipitation L3 1 day 0.25 degree x 0.25 degree V7, Edited by Andrey Savtchenko, Goddard Earth Sciences Data and Information Services Center (GES DISC) Accessed: 2017-03-01. Available from: `https://disc.gsfc.nasa.gov/datacollection/TRMM_3B42_Daily_7.html`.

2. Pastor-Escuredo D, Morales-Guzmán A, Torres-Fernández Y, Bauer JM, Wadhwa A, Castro-Correa C, et al. Flooding through the lens of mobile phone activity. In: Global Humanitarian Technology Conference (GHTC), 2014 IEEE. IEEE; 2014. p. 279–286.
